# Dating and relationship violence victimization and perpetration among 11–16 year olds in Wales: a cross-sectional analysis of the School Health Research Network (SHRN) survey

**DOI:** 10.1093/pubmed/fdz084

**Published:** 2019-08-29

**Authors:** Honor Young, Sara Jayne Long, G J Melendez-Torres, Hyun Sue Kim, Gillian Hewitt, Simon Murphy, Graham F Moore

**Affiliations:** DECIPHer, UKCRC Centre of Excellence, Cardiff University, 1-3 Museum Place, Cardiff, UK

**Keywords:** young people, violence, relationships

## Abstract

**Background:**

This study examines the prevalence of dating and relationship violence (DRV) victimization, perpetration and joint victimization and perpetration, and associations between DRV and socio-demographic characteristics.

**Methods:**

Cross-sectional self-report data from 74 908 students aged 11–16 from 193 schools across Wales were collected and analysed using generalized estimating equations to examine prevalence and predictors of emotional and physical DRV victimization, perpetration and joint victimization and perpetration.

**Results:**

More girls reported emotional victimization (28%) and perpetration (18%) than boys (20% and 16%, respectively). More girls (8%) than boys (7%) reported physical perpetration. However, boys (17%) reported more physical victimization than girls (12%). Age-related trajectories of DRV victimization and perpetration were stronger in girls than in boys. Students from single or step parent homes, those in care, and certain ethnic minority groups had increased odds of DRV. No association was found between socioeconomic status and DRV.

**Conclusions:**

Age-related trajectories and the lack of social patterning by socioeconomic status point to the value of early, universal interventions, while some evidence of ethnic patterning and family structure-related risk factors suggest areas for further research and targeted interventions. DRV continues to be a major public health problem for which little UK-specific intervention evidence exists.

## Introduction

Dating and relationship violence (DRV) encompasses threats, emotional abuse, coercion and controlling behaviours, physical violence, and coerced, non-consensual or abusive sexual activities.^1,2^ DRV includes behaviours associated with domestic violence (the term used by the UK government for psychological, sexual, emotional violence or abuse experienced by those aged 16 years or older) but is more frequently used for young people aged under 16, who are less likely to be living with their romantic partner. Internationally, 10–50% of women report some form of violence from current or previous partners.^3,4^ Most research focuses on adult populations; legal definitions for which individuals can be prosecuted for DRV apply only to individuals aged 16 or older, reflecting and reinforcing a perception of DRV as a problem only experienced in adult relationships.^1^ However, there is emerging evidence that DRV is experienced by children and adolescents, with early exposure related to later substance misuse, sexually transmitted infections (STIs) and teenage pregnancy,^5^ eating disorders, mental health problems, anti-social behaviour^6^ and violence.^7^ In 2008, domestic violence was estimated to cost the UK National Health Service (NHS) £1.73bn per year.^8^ However it is not known how much of this cost is attributed to those under 16 years old.

DRV represents a public health problem both in the UK and internationally, and there is growing recognition of its impacts for young people. In light of this, UK governments and WHO^9–11^ have requested new comprehensive DRV interventions for young people. The United Nations Convention on the Rights of the Child (UNCRC)^12^ states that governments should protect children and young people from all forms of physical or mental violence. Welsh Government’s ‘Rights of Children and Young Persons (Wales) Measure’^13^ details its responsibility and commitment to fulfil the UNCRC, and its articles are also encompassed within the Violence Against Women, Domestic Abuse and Sexual Violence (Wales) Act (2015)^10^, the Equality Act (2010)^14^ and the Well-being of Future Generations (Wales) Act (2015).^15^ Similar measures have been employed by statutory bodies in England to ensure compliance with the UNCRC.^12^

Despite some evidence of gender symmetry in mental health outcomes among DRV victims, research primarily focuses on females;^16^ less is known about the prevalence, causes and consequences of DRV exposure in males.^17^ Cross-sectional research in England and Wales found that among 16–19 year old females and males, 46% and 50%, respectively experienced controlling behaviours (e.g. told you who you could see or where you could go), while 32% and 27% reported threatening behaviours (e.g. threatened to hurt you physically).^18^ Other cross-sectional, although now dated, research from young people aged 13–17 from England, Scotland, and Wales found that DRV exposure was experienced by both boys and girls, although with clearer gender differences; 75% of girls vs 50% of boys reported experience of emotional violence, while 25% of girls vs 18% of boys reported experience of physical violence.^19^ In Europe, cross-sectional research in Germany found that 77% of 14-17 year olds had relationship experience and were therefore considered ‘at risk’ of DRV; 66% of female and 60% of male students with dating experience reported at least one kind of DRV (controlling behaviour, verbal aggression, coercion and threats operationalized as emotional violence).^20^

Evidence of associations between socio-demographic characteristics and exposure to DRV is equivocal. A review of 61 studies reported lower socio-economic status (SES) was associated with an increased risk for DRV victimization in adults.^21^ Other nationally representative population-based studies for women and girls report that DRV increases with deprivation,^22,23^ whereas others found no association.^24^ Few consistent associations have been identified between ethnicity and DRV victimization;^21^ some studies report no association^23,25^ and others higher rates among ethnic minority groups.^26^ Other non-nationally representative US research has found lower rates for adults^27^ and UK studies have reported higher rates of victimization for ethnic, adult minority groups.^21^ Nationally representative US research identified variation in the rates of DRV perpetration among ethnic minority groups.^28^

While evidence is emerging on adolescent exposure to DRV, reviews suggest perpetration rates among adolescents ranging from 14% to 81% for psychological and 11% to 46% for physical violence perpetration, although the definitions and operationalisation of DRV varies across studies, as do the samples and methods of data collection.^24,29,30^ A tendency to focus on exposure in isolation from perpetration, frames these experiences as unidirectional, potentially giving rise to differing implications for intervention than where a strong interaction between exposure and perpetration is present. One US study found 35% and 31% of 16 year olds reported DRV victimization and perpetration respectively, with significant correlation between the two^28,31^. No studies have examined perpetration, and its overlap with exposure to DRV, among young people in the UK. This overlap, particularly for physical violence, may in part reflect defensive behaviour in response to a physical threat or assault. However, reciprocal name-calling and hurtful comments within relationships may reflect a tendency for mutually conflict-filled relationships, rather than a clear distinction between victim and perpetrator. In contexts where these processes are unidirectional, or involve defensive violence, intervention may focus at least in part on supporting victims and punishing and reforming perpetrators. Where mutual conflict within young people’s relationships is more common, intervention focused more holistically on supporting young people in the development of healthy relationships may be indicated.

At present there are no nationally representative studies of young people’s experience of DRV in the UK. In this analysis, we address this gap, consider victimization, perpetration and joint victimization-perpetration, and additionally consider risk factors separately between genders. Hence, this paper will explore the following research questions:

1. What is the prevalence of DRV in young people aged 11 to 16 in Wales?

2. What is the socio-demographic patterning of victimization, perpetration and joint victimization-perpetration of DRV in young people aged 11 to 16 in Wales?

3. Does socio-demographic patterning of DRV differ between boys and girls aged 11 to 16 in Wales?

## Methods

Data were from the 2017 School Health Research Network (SHRN) Student Health and Wellbeing (SHW) survey. Further details are in Online Supplement 1. The SHW survey is an online, closed response, self-completion survey, available in English and Welsh. It measures self-reported health and wellbeing indicators among school students aged 11–16 years, and includes questions from the 2017/2018 Welsh Health Behaviour in School-aged Children (HBSC) survey with additional questions reflecting current policy, practice and research priorities in Wales. All network schools (*n* = 212) were invited to participate in the 2017 SHW survey between September and December. Dating and relationship questions were asked in all schools, but in 88 schools, they were only visible to approximately 40% of students as the remaining students were randomly allocated/routed to a different version of the survey. A total of *n* = 81,093 students were asked questions about dating and relationships.

## Measures

### Socio-demographic characteristics

Students indicated their sex, year and month of birth and year of study. Family socioeconomic status (SES) was measured using the Family Affluence Scale (FAS).^32^ Six survey items related to bedroom occupancy, car, computer and dishwasher ownership, family holidays, and number of bathrooms in the household were summed. Scores are recorded on a scale of 1–3; sum totals were split into tertiles. Ethnicity was asked using the following self-report categories: White British; White Irish, White Gypsy/Traveller; White Other; Mixed or Multiple Ethnic Group; Pakistani; Indian; Bangladeshi; Chinese; African; Caribbean or Black; Arab; Other; or I do not want to answer. Responses were categorized into; White British or Irish; White Traveller; White Other; Mixed Ethnicity or Other; South Asian (Pakistani, Indian, Bangladeshi), Chinese, African or Caribbean or Black, Arab. Household composition was measured by asking who participants lived with; Mother; Father; Mother’s partner; Father’s partner; Grandparent(s); Aunt(s)/uncle(s); Adult brothers and/or sisters; Foster parents; I live in residential care or a children’s home; I live independently (on my own or with friends or my partner); Someone or somewhere else or I do not want to answer. These were categorized as those living with: both parents; stepfamily; single mother; single father; foster care; or other.

### DRV measures

Participants were asked ‘have you ever been ‘seeing’, ‘dating’ or ‘going out with’ someone? Response options were ‘yes’, ‘no’, or ‘I don’t want to answer’. Those who responded ‘yes’ were asked about DRV victimization and perpetration. Based on Barter *et al.*’s (2009) research^19^, participants were given the following statements to respond to: ‘a partner has made hurtful comments towards me’, ‘a partner has pushed, shoved or slapped me’ and ‘a partner has punched or kicked or beat me up’, ‘I have made hurtful comments to a partner,’ ‘I have pushed shoved or slapped a partner’, ‘I have punched or kicked or beat up a partner’. Response options included ‘never’, ‘once’, ‘a few times’, ‘often’, and ‘I do not want to answer’. Responses were combined to provide binary indicators of ever exposure to and perpetration of emotional and physical violence.

### Statistical analyses

We undertook all models within a generalized estimating equations (GEE) framework to provide a population-average coefficient while addressing school-level clustering. We first estimated the relationship between gender and each form of DRV perpetration, victimization or joint perpetration-victimization. We then entered all socio-demographic predictors into models for each form of DRV simultaneously, stratifying by gender. Finally, we checked for differences in risk factor patterns by interacting gender with each predictor one at a time in fully adjusted, unstratified models. The denominator in the main models presented is boys or girls reporting any dating experience. All models were estimated using a logit link, an exchangeable correlation matrix and Huber-White robust standard errors accounting for school-level clustering. Models were estimated in Stata v.14 (Statacorp, College Station, TX).

### Research ethics and consent

Ethical approval was granted by Cardiff University School of Social Sciences Research Ethics Committee. Further details are in Online Supplement 1.

## Results

Data were collected from 193 schools from 74 908 students. Sample characteristics are detailed in Online Supplements 2, 3 and 4. The sample consisted of approximately even numbers of boys and girls. The majority reported their ethnicity as White British or Irish. Two-thirds reported living with both parents, around 15% reported living with a single mother, or with a parent and step-parent. Over half reported dating experience.

### Gender differences in DRV exposure and perpetration

Overall, significantly more girls (28%) with dating experience reported emotional victimization than boys (20%) (Table 1) (OR=1.52, 95% CI [1.44, 1.60]). Similarly, more girls (18%) reported emotional perpetration than boys (16%; OR=1.17, 95% CI [1.10, 1.24]). Girls also reported more physical perpetration (8%) than boys (7%; OR=1.27, 95% CI [1.17, 1.39]) (Table 2). Conversely more boys (17%) reported experience of physical victimization, which was reported by up to 12% of girls (OR=0.67, 95% CI [0.63, 0.71]). More girls (17%) reported dual emotional victimization and perpetration than boys (13%; OR=1.28, 95% CI [1.21, 1.37]). However, there was no significant sex difference in prevalence of dual physical victimization and perpetration, at 6% for both boys and girls (OR=1.04, 95% CI [0.96, 1.14]).

**Table 1:** Prevalence of emotional victimization, perpetration and sociodemographic characteristics for the sample of 11–16 year olds with dating experience in Wales

	Emotional victimization % (*n*)		Emotional perpetration % (*n*)		Emotional victimization and perpetration % (*n*)	
	Boys	Girls	Boys	Girls	Boys	Girls
**Overall**	20.1 (3915/19 478)	27.6 (5565/20 172)	16.2 (3158/19 475)	18.4 (3721/20 210)	13.4 (2594/19 431)	16.5 (3320/20 137)
**Year**						
**7**	15.2 (543/3566)	15.9 (521/3288)	9.2 (327/3561)	7.6 (250/3289)	7.3 (259/3552)	6.2 (204/3278)
**8**	15.9 (642/4041)	19.3 (791/4096)	12.1 (488/4040)	10.4 (428/4098)	9.6 (385/4028)	8.7 (357/4083)
**9**	18.7 (810/4340)	26.8 (1210/4512)	15.3 (663/4340)	17.6 (797/4532)	12.3 (534/4330)	15.7 (705/4506)
**10**	23.1 (907/3925)	32.9 (1368/4165)	19.6 (770/3924)	23.2 (966/4173)	16.1 (630/3916)	21.0 (875/4161)
**11**	28.1 (1013/3606)	40.7 (1675/4111)	25.2 (910/3610)	31.1 (1280/4118)	21.8 (786/3605)	28.7 (1179/4109)
**FAS**						
**Low**	20.6 (1292/6265)	28.2 (1960/6963)	16.9 (1058/6260)	19.2 (1336/6970)	13.9 (867/6251)	17.1 (1185/6949)
**Medium**	20.6 (1262/6113)	27.1 (1705/6298)	16.5 (1008/6112)	18.4 (1164/6316)	13.7 (836/6094)	16.5 (1036/6286)
**High**	19.2 (1361/7100)	27.5 (1900/6911)	15.4 (1092/7103)	17.6 (1221/6924)	12.6 (891/7086)	15.9 (1099/6902)
**Ethnicity**						
**White British or Irish**	19.4 (3265/16 833)	27.5 (4931/17 933)	15.4 (2595/16 833)	18.1 (3258/17 969)	12.7 (2131/16 795)	16.4 (2929/17 906)
**White Traveller**	38.8 (73/188)	48.2 (53/110)	31.4 (59/188)	39.1 (43/110)	29.3 (55/188)	31.8 (35/110)
**White Other**	20.5 (117/571)	28.9 (155/536)	15.9 (90/566)	19.0 (102/538)	13.6 (77/566)	15.9 (85/535)
**Mixed Ethnicity or Other**	22.6 (185/817)	28.5 (227/798)	18.5 (152/820)	20.1 (161/800)	14.2 (116/816)	17.3 (138/796)
**South Asian (Pakistani, Indian, Bangladeshi)**	25.3 (66/261)	29.9 (52/174)	28.0 (73/261)	26.0 (45/173)	22.7 (59/260)	23.8 (41/172)
**Chinese**	28.2 (20/71)	29.3 (17/58)	22.9 (16/70)	21.1 (12/57)	20.0 (14/70)	17.5 (10/57)
**African or Caribbean or Black**	24.7 (62/251)	25.4 (32/126)	25.9 (65/251)	23.8 (30/126)	20.7 (52/251)	18.3 (23/126)
**Arab**	37.1 (43/116)	20.0 (11/55)	34.5 (40/116)	23.6 (13/55)	30.2 (35/116)	20.0 (11/55)
**Family structure**						
**Both parents**	17.9 (2043/11 431)	24.8 (2848/11 474)	14.2 (1619/11 424)	16.1 (1849/11 490)	11.7 (1332/11 410)	14.3 (1638/11 458)
**Single mum**	22.0 (671/3050)	30.3 (1107/3651)	18.0 (550/3057)	21.0 (769/3662)	14.7 (448/3049)	18.7 (681/3644)
**Single dad**	24.5 (109/445)	37.0 (150/405)	21.0 (93/444)	28.6 (116/405)	16.9 (75/443)	26.9 (108/402)
**Parent & Step-Parent**	23.2 (625/2700)	31.7 (1087/3425)	18.1 (488/2701)	20.6 (707/3437)	15.2 (410/2690)	18.9 (648/3424)
**Care**	32.8 (88/268)	41.0 (96/234)	36.3 (97/267)	35.5 (82/231)	27.0 (72/267)	30.3 (70/231)
**Other**	21.2 (14/66)	25.8 (8/31)	18.5 (12/65)	16.1 (5/31)	13.9 (9/65)	16.1 (5/31)

**Table 2:** Prevalence of physical victimization, perpetration and sociodemographic characteristics for the sample of 11-16 year olds with dating experience in Wales

	Physical victimization % (*n*)		Physical perpetration % (*n*)		Physical victimization and perpetration % (*n*)	
	Boys	Girls	Boys	Girls	Boys	Girls
**Overall**	17.3 (3369/19 481)	12.3 (2469/20 158)	6.7 (1311/19 493)	8.4 (1704/20 221)	6.1 (1176/19.454)	6.3 (1267/20 136)
**Year**						
**7**	16.9 (602/3570)	9.7 (320/3285)	6.2 (222/3562)	5.9 (194/3293)	5.2 (186/3558)	4.3 (142/3281)
**8**	15.1 (610/4048)	9.2 (378/4098)	5.6 (225/4057)	6.1 (249/4107)	5.0 (202/4045)	4.4 (178/4091)
**9**	16.6 (719/4338)	11.8 (533/4509)	6.5 (280/4340)	8.2 (373/4535)	5.7 (245/4331)	6.1 (276/4506)
**10**	18.1 (708/3915)	14.4 (597/4160)	7.7 (300/3921)	10.0 (415/4172)	7.1 (277/3911)	7.5 (311/4157)
**11**	20.2 (730/3610)	15.6 (641/4106)	7.9 (284/3613)	11.5 (473/4114)	7.4 (266/3609)	8.8 (360/4101)
**FAS**						
**Low**	18.1 (1133/6266)	13.1 (912/6956)	7.0 (440/6266)	9.1 (636/6982)	6.3 (391/6257)	6.7 (467/6950)
**Medium**	17.9 (1097/6114)	12.1 (762/6296)	7.0 (426/6122)	8.4 (529/6316)	6.3 (382/6105)	6.4 (404/6285)
**High**	16.0 (1139/7101)	11.5 (795/6906)	6.3 (445/7105)	7.8 (539/6923)	5.7 (403/7092)	5.7 (396/6901)
**Ethnicity**						
**White British or Irish**	16.4 (2762/16 839)	11.9 (2138/17 921)	5.9 (994/16 854)	8.1 (1459/17 975)	5.3 (894/16 820)	6.0 (1081/17 904)
**White Traveller**	38.2 (71/186)	29.1 (32/110)	23.5 (44/187)	24.6 (27/110)	22.0 (41/186)	18.2 (20/110)
**White Other**	17.9 (102/569)	14.0 (75/535)	6.7 (38/568)	8.8 (47/535)	5.8 (33/567)	6.6 (35/533)
**Mixed Ethnicity or Other**	19.4 (159/820)	13.9 (111/800)	9.3 (76/819)	9.2 (74/803)	8.3 (68/819)	7.0 (56/799)
**South Asian (Pakistani, Indian, Bangladeshi)**	25.3 (66/261)	13.2 (23/174)	18.8 (49/261)	10.9 (19/175)	17.7 (46/260)	8.7 (15/173)
**Chinese**	26.1 (18/69)	14.0 (8/57)	14.7 (10/68)	8.8 (5/57)	10.3 (7/68)	8.8 (5/57)
**African or Caribbean or Black**	26.3 (66/251)	13.4 (17/127)	15.1 (38/251)	15.0 (19/127)	13.9 (35/251)	10.2 (13/127)
**Arab**	33.6 (39/116)	20.0 (11/55)	22.4 (26/116)	20.0 (11/55)	19.8 (23/116)	20.0 (11/55)
**Family structure**						
**Both parents**	14.6 (1663/11 428)	10.1 (1160/11 463)	5.3 (603/11 436)	6.9 (798/11 499)	4.7 (536/11 416)	5.0 (572/11 455)
**Single mum**	18.7 (571/3049)	13.5 (493/3656)	6.7 (205/3051)	9.7 (354/3669)	6.1 (185/3046)	7.4 (269/3653)
**Single dad**	21.4 (95/445)	16.5 (66/401)	9.7 (43/444)	13.3 (54/407)	8.6 (38/444)	9.2 (37/401)
**Parent & Step-Parent**	20.3 (548/2701)	14.5 (498/3424)	6.3 (170/2704)	9.0 (309/3430)	5.6 (152/2698)	6.8 (231/3417)
**Care**	39.8 (107/269)	31.3 (72/230)	32.8 (88/268)	26.4 (61/231)	31.0 (83/268)	23.0 (53/230)
**Other**	24.2 (16/66)	16.1 (5/31)	18.5 (12/65)	16.1 (5/31)	13.9 (9/65)	12.9 (4/31)

### Socio-demographic patterning in DRV victimization and perpetration

For girls and boys, increasing age was associated with steadily greater odds of victimization, perpetration and joint victimization-perpetration of both emotional DRV (Table 3) and physical DRV (Table 4). This pattern was weakest for physical DRV perpetration reported by boys. No consistent association was found between family SES and DRV victimization or perpetration. Compared to students from families with both parents, students from single or step parent homes and those in care were at increased odds of reporting some form of DRV victimization or perpetration. Relationships were especially pronounced for physical DRV victimization, perpetration and joint victimization-perpetration in children residing in care. Boys and girls from certain ethnic minority groups had greater odds of DRV victimization and perpetration than White British or Irish ethnicities.

**Table 3:** Adjusted odds ratios (95% confidence intervals) for the association between emotional victimization, perpetration and both victimization and perpetration and sociodemographic characteristics for 11–16 year olds with dating experience in Wales

	Emotional victimization % (*n*)			Emotional perpetration % (*n*)			Emotional victimization and perpetration % (*n*)		
	Boys (*n* = 17 664)	Girls (*n* = 18 890)	Interaction	Boys (*n* = 17 662)	Girls (*n* = 18 926)	Interaction	Boys (*n* = 17 629)	Girls (*n* = 18 861)	Interaction
**Year**									
**7**	*1 (Ref)*	*1 (Ref)*	*1 (Ref)*	*1 (Ref)*	*1 (Ref)*	*1 (Ref)*	*1 (Ref)*	*1 (Ref)*	*1 (Ref)*
**8**	1.05 (0.90–1.22)	1.30 (1.13–1.50)***	1.24 (0.99–1.54)	1.34 (1.14–1.58)***	1.48 (1.23–1.76)***	1.10 (0.86–1.40)	1.32 (1.10–1.58)**	1.46 (1.19–1.80)***	1.11 (0.84–1.47)
**9**	1.28 (1.11–1.47)**	2.00 (1.75–2.29)***	1.55 (1.27–1.89)***	1.76 (1.51–2.05)***	2.68 (2.24–3.21)***	1.51 (1.19–1.93)**	1.77 (1.49–2.12)***	2.83 (2.32–3.46)***	1.58 (1.20–2.09)**
**10**	1.64 (1.44–1.88)***	2.66 (2.36–3.00)***	1.59 (1.33–1.91)***	2.35 (2.03–2.71)***	3.72 (3.19–4.33)***	1.59 (1.31–1.92)***	2.37 (2.01–2.79)***	4.01 (3.37–4.76)***	1.68 (1.34–2.11)***
**11**	2.15 (1.86–2.49)***	3.76 (3.27–4.32)***	1.70 (1.39–2.08)***	3.32 (2.88–3.82)***	5.76 (4.90–6.78)***	1.71 (1.40–2.07)***	3.50 (2.95–4.15)***	6.20 (5.19–7.41)***	1.74 (1.37–2.20)***
**FAS**									
**Low**	*1 (Ref)*	*1 (Ref)*	*1 (Ref)*	*1 (Ref)*	*1 (Ref)*	*1 (Ref)*	*1 (Ref)*	*1 (Ref)*	*1 (Ref)*
**Medium**	1.02 (0.94–1.11)	0.96 (0.88–1.04)	0.93 (0.84–1.04)	0.97 (0.88–1.08)	0.99 (0.90–1.08)	1.00 (0.88–1.15)	1.00 (0.89–1.12)	0.99 (0.89–1.09)	0.98 (0.85–1.31)
**High**	0.96 (0.88–1.06)	1.02 (0.93–1.12)	1.05 (0.93–1.18)	0.93 (0.84–1.03)	0.96 (0.87–1.06)	1.01 (0.89–1.14)	0.94 (0.83–1.05)	0.98 (0.87–1.09)	1.02 (0.89–1.16)
**Ethnicity**									
**White British or Irish**	*1 (Ref)*	*1 (Ref)*	*1 (Ref)*	*1 (Ref)*	*1 (Ref)*	*1 (Ref)*	*1 (Ref)*	*1 (Ref)*	*1 (Ref)*
**White Traveller**	2.25 (1.55–3.26)***	2.24 (1.48–3.39)***	1.03 (0.59–1.78)	1.92 (1.29–2.85)**	2.64 (1.66–4.19)***	1.40 (0.76–2.59)	2.25 (1.50–3.38)***	2.16 (1.44–3.24)***	1.00 (0.54–1.82)
**White Other**	1.16 (0.94–1.44)	1.15 (0.96–1.37)	0.97 (0.74–1.26)	1.09 (0.86–1.38)	1.12 (0.90–1.39)	1.02 (0.73–1.41)	1.17 (0.89–1.54)	1.02 (0.82–1.26)	0.85 (0.60–1.20)
**Mixed Ethnicity or Other**	1.23 (1.01–1.51)*	1.19 (1.03–1.37)*	0.94 (0.72–1.22)	1.25 (1.01–1.54)*	1.29 (1.06–1.56)*	1.00 (0.76–1.31)	1.14 (0.91–1.44)	1.22 (1.01–1.48)*	1.04 (0.78–1.39)
**South Asian (Pakistani, Indian, Bangladeshi)**	1.36 (1.02–1.82)*	1.09 (0.75–1.57)	0.82 (0.50–1.36)	1.99 (1.45–2.73***	1.50 (1.02–2.21)*	0.76 (0.43–1.33)	1.91 (1.40–2.61)***	1.49 (0.96–2.29)	0.79 (0.43–1.48)
**Chinese**	1.36 (0.77–2.38)	1.27 (0.70–2.32)	0.90 (0.40–2.05)	1.37 (0.70–2.66)	1.43 (0.70–2.92)	0.98 (0.38–2.49)	1.39 (0.71–2.72)	1.29 (0.60–2.77)	0.89 (0.33–2.43)
**African or Caribbean or Black**	1.23 (0.87–1.72)	0.83 (0.57–1.23)	0.69 (0.43–1.09)	1.68 (1.17–2.42)**	1.32 (0.81–2.15)	0.78 (0.48–1.27)	1.59 (1.08–2.34)*	1.02 (0.62–1.68)	0.65 (0.38–1.09)
**Arab**	2.28 (1.51–3.43)***	0.72 (0.31–1.70)	0.33 (0.13–0.80)*	2.43 (1.56–3.78)***	1.64 (0.72–3.73)	0.68 (0.27–1.68)	2.66 (1.74–4.07)***	1.47 (0.60–3.59)	0.56 (0.21–1.50)
**Family structure**									
**Both parents**	*1 (Ref)*	*1 (Ref)*	*1 (Ref)*	*1 (Ref)*	*1 (Ref)*	*1 (Ref)*	*1 (Ref)*	*1 (Ref)*	*1 (Ref)*
**Single mum**	1.24 (1.12–1.37)***	1.30 (1.19–1.41)***	1.04 (0.92–1.19)	1.24 (1.11–1.39)***	1.33 (1.20–1.48)***	1.08 (0.92–1.26)	1.23 (1.09–1.38)***	1.33 (1.20–1.48)***	1.09 (0.93–1.27)
**Single dad**	1.41 (1.13–1.75)**	1.59 (1.26–2.01)***	1.15 (0.82–1.60)	1.47 (1.17–1.84)**	1.78 (1.42–2.23)***	1.24 (0.88–1.73)	1.39 (1.08–1.80)*	1.89 (1.50–2.39)***	1.38 (0.97–1.94)
**Parent & Step–Parent**	1.38 (1.24–1.53)***	1.44 (1.32–1.57)***	1.04 (0.91–1.19)	1.32 (1.17–1.49)***	1.37 (1.24–1.52)***	1.04 (0.88–1.21)	1.35 (1.18–1.54)***	1.43 (1.29–1.59)***	1.06 (0.90–1.26)
**Care**	2.10 (1.61–2.74)***	2.14 (1.62–2.83)***	0.98 (0.68–1.40)	3.16 (2.40–4.16)***	2.89 (2.12–3.96)***	0.89 (0.60–1.31)	2.51 (1.31–1.90)***	2.67 (1.96–3.64)***	1.02 (0.68–1.54)
**Other**	1.19 (0.61–2.31)	1.16 (0.54–2.49)	0.93 (0.34–2.55)	1.31 (0.65–2.64)	1.06 (0.44–2.60)	0.82 (0.27–2.44)	1.16 (0.50–2.70)	1.29 (0.53–3.14)	1.09 (0.34–3.50)

OR = odds ratio; Ref = reference. Statistically significant differences *P < 0.05; **P < 0.01, ***P < 0.001.

**Table 4:** Adjusted odds ratios (95% confidence intervals) for the association between physical victimization, perpetration and both victimization and perpetration and sociodemographic characteristics for 11–16 year olds with dating experience in Wales

	Physical victimization % (*n*)			Physical Perpetration % (*n*)			Physical victimization and perpetration % (*n*)		
	Boys (*n* = 17 661)	Girls (*n* = 18 878)	Interaction	Boys (*n* = 17 671)	Girls (*n* = 18 936)	Interaction	Boys (*n* = 17 641)	Girls (*n* = 18 861)	Interaction
**Year**									
**7**	*1 (Ref)*	*1 (Ref)*	*1 (Ref)*	*1 (Ref)*	*1 (Ref)*	*1 (Ref)*	*1 (Ref)*	*1 (Ref)*	*1 (Ref)*
**8**	0.89 (0.77–1.03)	0.98 (0.80–1.19)	1.09 (0.86–1.38)	0.86 (0.70–1.07)	1.13 (0.92–1.39)	1.33 (1.01–1.76)*	0.94 (0.74–1.19)	1.09 (0.85–1.40)	1.18 (0.85–1.64)
**9**	0.95 (0.82–1.09)	1.28 (1.06–1.54)*	1.36 (1.07–1.72)*	0.94 (0.76–1.16)	1.50 (1.23–1.84)***	1.61 (1.22–2.12)**	1.00 (0.79–1.25)	1.52 (1.19–1.94)**	1.55 (1.11–2.16)*
**10**	1.08 (0.95–1.24)	1.59 (1.35–1.89)***	1.48 (1.19–1.84)***	1.13 (0.92–1.38)	1.83 (1.52–2.20)***	1.65 (1.26–2.15)***	1.26 (1.03–1.56)*	1.88 (1.50–2.37)***	1.50 (1.11–2.04)**
**11**	1.26 (1.08–1.47)**	1.73 (1.42–2.10)***	1.38 (1.08–1.76)*	1.23 (0.99–1.54)	2.17 (1.75–2.69)***	1.78 (1.33–2.38)***	1.41 (1.11–1.80)**	2.21 (1.73–2.83)***	1.58 (1.14–2.18)**
**FAS**									
**Low**	*1 (Ref)*	*1 (Ref)*	*1 (Ref)*	*1 (Ref)*	*1 (Ref)*	*1 (Ref)*	*1 (Ref)*	*1 (Ref)*	*1 (Ref)*
**Medium**	1.03 (0.92–1.14)	0.93 (0.83–1.05)	0.91 (0.79–1.06)	0.98 (0.85–1.14)	0.94 (0.83–1.07)	0.95 (0.79–1.14)	0.99 (0.85–1.15)	0.99 (0.86–1.15)	0.99 (0.80–1.21)
**High**	0.93 (0.85–1.03)	0.94 (0.83–1.06)	1.00 (0.87–1.16)	0.92 (0.80–1.07)	0.92 (0.80–1.06)	0.97 (0.80–1.18)	0.96 (0.83–1.12)	0.95 (0.82–1.11)	0.96 (0.78–1.18)
**Ethnicity**									
**White British or Irish**	*1 (Ref)*	*1 (Ref)*	*1 (Ref)*	*1 (Ref)*	*1 (Ref)*	*1 (Ref)*	*1 (Ref)*	*1 (Ref)*	*1 (Ref)*
**White Traveller**	2.57 (1.81–3.65)***	2.77 (1.63–4.70)***	1.11 (0.59–2.08)	3.02 (1.97–4.63)***	3.41 (1.98–5.89)***	1.11 (0.57–2.16)	3.25 (2.11–5.01)***	3.08 (1.69–5.60)***	0.94 (0.45–1.97)
**White Other**	1.20 (0.96–1.49)	1.21 (0.93–1.58)	1.01 (0.72–1.40)	1.17 (0.83–1.67)	1.08 (0.81–1.43)	0.89 (0.57–1.41)	1.16 (0.82–1.66)	1.11 (0.78–1.57)	0.93 (0.56–1.53)
**Mixed Ethnicity or Other**	1.23 (1.00–1.51)*	1.26 (1.04–1.51)*	1.01 (0.76–1.34)	1.56 (1.19–2.05)**	1.16 (0.91–1.47)	0.72 (0.50–1.04)	1.56 (1.17–2.08)**	1.26 (0.98–1.62)	0.79 (0.56–1.13)
**South Asian (Pakistani, Indian, Bangladeshi)**	1.43 (1.07–1.93)*	1.05 (0.70–1.57)	0.72 (0.44–1.67)	2.86 (1.90–4.32)***	1.28 (0.82–2.00)	0.42 (0.24–0.73)**	3.03 (2.01–4.55)***	1.32 (0.81–2.15)	0.41 (0.22–0.77)**
**Chinese**	1.73 (0.99–3.04)	1.08 (0.55–2.14)	0.63 (0.27–1.46)	2.42 (1.15–5.09)*	0.86 (0.35–2.11)	0.32 (0.10–0.96)*	1.59 (0.61–4.19)	1.19 (0.49–2.89)	0.67 (0.20–2.21)
**African or Caribbean or Black**	1.42 (1.00–2.01)	1.00 (0.61–1.65)	0.71 (0.38–1.31)	1.99 (1.34–2.98)**	1.76 (1.05–2.94)*	0.86 (0.45–1.66)	2.03 (1.34–3.10)**	1.46 (0.85–2.51)	0.71 (0.35–1.41)
**Arab**	2.29 (1.46–3.58)***	2.04 (0.91–4.58)	0.89 (0.33–2.39)	3.71 (2.19–6.29)***	3.10 (1.37–7.02)**	0.80 (0.29–2.19)	3.33 (1.96–5.67)***	4.36 (1.90–10.02)**	1.25 (0.42–3.74)
**Family structure**									
**Both parents**	*1 (Ref)*	*1 (Ref)*	*1 (Ref)*	*1 (Ref)*	*1 (Ref)*	*1 (Ref)*	*1 (Ref)*	*1 (Ref)*	*1 (Ref)*
**Single mum**	1.30 (1.16–1.45)***	1.34 (1.20–1.48)***	1.04 (0.89–1.21)	1.21 (1.02–1.43)*	1.37 (1.19–1.58)***	1.16 (0.95–1.42)	1.24 (1.03–1.49)*	1.46 (1.24–1.72)***	1.20 (0.95–1.50)
**Single dad**	1.49 (1.19–1.88)**	1.62 (1.21–2.17)**	1.11 (0.74–1.67)	1.76 (1.27–2.43)**	1.75 (1.26–2.43)**	1.04 (0.66–1.64)	1.73 (1.21–2.47)**	1.72 (1.20–2.48)*	1.04 (0.62–1.74)
**Parent & Step–Parent**	1.47 (1.33–1.64)***	1.52 (1.35–1.72)***	1.03 (0.89–1.21)	1.21 (1.01–1.44)*	1.33 (1.15–1.54)***	1.12 (0.90–1.39)	1.23 (1.02–1.48)*	1.40 (1.19–1.65)***	1.16 (0.91–1.46)
**Care**	3.75 (2.85–4.94)***	3.90 (2.81–5.42)***	1.02 (0.68–1.51)	7.57 (5.64–10.16)***	4.49 (3.32–6.06)***	0.57 (0.38–0.87)**	8.00 (5.96–10.74)***	5.36 (3.77–7.64)***	0.65 (0.43–1.00)
**Other**	1.80 (0.94–3.46)	1.67 (0.58–4.75)	0.91 (0.26–3.15)	3.82 (1.89–7.72)***	2.60 (0.95–7.12)	0.66 (0.20–2.23)	3.05 (1.37–6.80)**	2.86 (0.86–9.48)	0.91 (0.23–3.68)

OR = odds ratio; Ref = reference. Statistically significant differences *P < 0.05; **P < 0.01, ***P < 0.001.

### Overlap between victimization and perpetration

As seen in Tables 1 and 2, the prevalence of joint victimization-perpetration for both emotional and physical DRV increased throughout adolescence. Of note is that percentages of joint victimization-perpetration were in each socio-demographic category closer to percentages of perpetration, suggesting that in the substantial majority of cases, those reporting perpetration also report victimization. Conversely, boys from Traveller groups and young people in care appear to experience physical victimization bi-directionally; that is, most victims are also perpetrators. Socio-demographic patterning for joint perpetration-victimization largely mirrored findings for either victimization or perpetration alone (see Tables 3 and 4). Compared to students from families with both parents, students from single or step parent homes, or those in care were at increased odds of reporting joint DRV victimization and perpetration. Boys and girls from certain ethnic minority groups were at increased odds of joint DRV victimization and perpetration compared to White British or Irish ethnicities.

### Socio-demographic patterning of DRV by gender

Interaction tests for each predictor showed where relationships between socio-demographic characteristics and DRV were different by gender. As seen by significant interaction terms, girls have a faster increase by grade in all forms of DRV victimization or perpetration. For example, against steadily increasing odds of boys reporting emotional victimization by age, the difference between boys’ and girls’ increase in reporting from year 7 to year 8 is OR=1.24, 95% CI (0.99, 1.54) whereas the difference between boys’ and girls’ increase in reporting from year 7 to year 11 is OR=1.70, 95% CI (1.39, 2.08). In the case of physical victimization and perpetration, where boys’ trajectories of reporting are less clear, significant interaction terms confirm that a trajectory of increasing prevalence exists for girls that does not exist for boys. Findings for moderation of the relationship between ethnicity and DRV were largely null, though occasionally indicated that girls were less likely to report victimization or perpetration of DRV as compared to boys reporting the same ethnicity. Finally, interaction terms for family structure were largely non-significant but indicated that living in care was a less strong risk factor for physical DRV perpetration for girls than for boys (OR=0.57, 95% CI [0.38, 0.87]).

## Discussion

### Main finding of this study

We reported the prevalence of physical and emotional DRV perpetration, victimization and joint perpetration-victimization, considered the socio-demographic patterning of DRV, and examined if this patterning differed by gender. No consistent association was found between family SES and DRV. Older age was associated with increased odds of emotional victimization, perpetration and joint victimization-perpetration. Similar patterning was found for physical DRV among girls, but was less clear among boys. Girls reported a faster increase in prevalence of DRV with increasing grade as compared to boys. Students from single or step parent homes, and those in care, as well as certain ethnic minority groups were at increased odds of DRV.

### What is already known on this topic?

Existing UK cross-sectional research suggests that up to 75% of girls and 50% of boys report emotional and 25% of girls and 18% of boys report physical victimization.^19^ Estimates of DRV perpetration reach 81% for psychological and 46% for physical perpetration.^24,29,30^ Existing research with older adolescents suggests around 80% of boys and girls experience bidirectional verbal or emotional violence.^33,34^ Figures for bidirectional physical violence vary.^35^ Among US high school students (mean age 16.63 years), 35% reportedly respond to DRV victimization with perpetration; teenagers most common responses to physical aggression in a relationship were aggressive action, informal help seeking, threatened or actual breakup, and doing nothing (males) or crying (females). Females were more likely to fight back than males.^36^ Evidence of associations between socio-demographic characteristics and DRV victimization is equivocal; there is no clear, consistent patterning relating to SES and minority ethnic groups.^21,23–26,28^ While there is dearth of literature on young people’s experience of DRV, this is especially the case in relation to combined DRV exposure and perpetration. Existing research suggests that there is a correlation between the two.^28,31^

### What this study adds

This is the first study to provide a profile of DRV from a nationally representative sample of young people aged 11–16 years old in Wales. Our estimates of DRV victimization and perpetration are lower than other cross-sectional estimates from the UK and Europe.^18–20,24,30^ Our sample includes younger adolescents than other research which may explain the lower estimates (fewer younger students report dating experience and therefore DRV.

More girls reported emotional DRV victimization than boys, but physical victimization was experienced more by boys than girls. At younger ages, rates were similar; as adolescents got older, girls reported more perpetration of emotional and physical violence. A possible explanation is social desirability bias. Culturally, there is less social tolerance of violence perpetration by men;^37,38^ if a man hits a women it is usually considered less acceptable than if a woman hits a man.^39^ Hence, boys who have perpetrated DRV may be less likely to report this truthfully. It may also be the case that the gender of the victim is important in the social acceptability of behaviour. That is, it is perhaps violence towards males which is more socially tolerated generally, rather than violence by women being more tolerated. Interventions are needed which are effective for both males and female adolescents in reducing perpetration and victimization of DRV.

The lack of social patterning of DRV despite the large sample is consistent with existing literature which has found no evidence that DRV is socially stratified.^24^ This highlights the importance of the universal, primary prevention of DRV since these behaviours are widespread. SES was measured using the FAS; more research is required exploring other key indicators of DRV for example other measures of SES.

We observed age-related trajectories of DRV victimization and perpetration. Whether this is due to an increase in dating experience (thus increased risk of DRV over multiple relationships) or increased awareness or acceptability of these behaviours is unclear. However, our findings highlight the need for early intervention given the lifecourse consequences of DRV. Longitudinal research suggests that once victimized, young people were at increased risk for cumulative revictimisation later in adolescence.^40^ Early intervention can establish positive relationship norms and prevent negative developmental cascades arising from early experience of DRV.

While universal interventions are helpful, targeted interventions can address those at greatest risk of DRV. At present there is a lack of evidence for successful DRV interventions in the UK. Young people from single parent or step parent families and especially those in care reported higher odds of victimization, perpetration and combined victimization and perpetration. These findings are consistent with existing international literature which suggests that adverse relationships with caregivers may negatively influence subsequent relationships.^41–46^ Some of the primary reasons for referral to social services include domestic abuse, family dysfunction and family stress.^47^ Young people who experience adversity and children in care^48,49^ are at increased risk of becoming a perpetrator and victim of DRV.^50–53^ The present study also found that certain ethnic minority groups experienced greater odds of DRV. Existing research has linked ethnic minority group differences in DRV to other factors related to ethnicity, such as SES.^54^ We were able to control for SES in our analyses, suggesting some residual association between ethnicity and DRV warranting further investigation.

One key strength of the paper is the disaggregation of emotional and physical victimization. The degree of overlap between victimization and perpetration for emotional violence seems to increase throughout adolescence (especially for boys). Physical violence appears to be largely unidirectional, whereas emotional violence tended to be bidirectional. These figures were similar to existing literature.^33–36^ The higher rates of bidirectional violence may reflect greater normalization of verbal and emotional abuse in relationships relative to physical violence. Further research is needed to extend gender symmetry/asymmetry accounts of bidirectional intimate partner violence to the DRV context.

### Limitations of this study

The prevalence of DRV reported may be an underrepresentation due to the subjective nature of responding, and stigma associated with being a victim or perpetrator of DRV. While comparison to existing literature is key, it is limited by the changing definitions of DRV. At present, measurement of DRV is limited to emotional and physical violence, and does not include sexual violence or violence through forms of technology (e.g. social media), nor does it distinguish the severity of DRV. Although DRV is correlated with other indices, the cross-sectional design means that causality cannot be established. Similarly, nothing is known about the circumstances and context of young people’s dating and relationships; for example, we do not know the sex/gendered nature of young people’s relationships, nor do we know the circumstances of the violence. For example, bidirectional violence may reflect a combination of mutually conflict–filled relationships, and defensive behaviour in response to violence, and while these issues have different implications for intervention they cannot be easily disentangled. More nuanced questioning is required to disentangle the contexts of young people’s dating and relationship behaviours, and the associations between these contexts, bidirectional conflict and joint victimization-perpetration.

## Supplementary Material

fdz084_Online_supplement_1

fdz084_Online_supplement_2

fdz084_Online_supplement_3

fdz084_Online_supplement_4
