# Supplementary material for: Dating and relationship violence victimization and perpetration among 11–16 year olds in Wales: a cross-sectional analysis of the School Health Research Network (SHRN) survey
Source: J Public Health (Oxf). 2019 Aug 29;43(1):111–22. doi: 10.1093/pubmed/fdz084 (PMC8042367; doi:10.1093/pubmed/fdz084)
Supplement: fdz084_Online_supplement_1 [file fdz084_online_supplement_1.docx]

**Online supplement 1: The School Health Research Network (SHRN) Student Health and Wellbeing (SHW) survey**

The School Health Research Network (SHRN) is a multiagency partnership led by the Centre for the Development and Evaluation of Complex Interventions for Public Health Improvement (DECIPHer) at Cardiff University; with Welsh Government, Public Health Wales, Cancer Research UK and as of October 2017, 212 secondary schools. At the time of the 2017 survey, all maintained secondary schools in Wales were members. Schools joined the Network in three ways: those participating in the Welsh Health Behaviour in School-aged Children (HBSC) survey in 2013/2014 were invited (60 out of 82 joined); nine schools in South Wales recruited to an HBSC sub-study to pilot data linkage methods joined; and two rounds of open recruitment.

Schools returned a registration form indicating their intention to participate in the study. Schools informed parents about the survey using two of three methods (letters sent home with students or via email, and a text message notification about the letter) and parents had the option of withdrawing their child from data collection (‘opt-out’ consent procedure). The survey was voluntary and completed anonymously by the majority of students. In 39 schools, students were asked to give some identifying information, including their name, at the end of the survey as part of a data linkage pilot project.

Students were informed that their identifying information would be stored separately to their survey responses, used for research only and never used to identify their responses. Only the fully anonymised data are used for the analyses in this paper. The first question of the survey asked students for their consent to participate and if they said no, the survey automatically closed. Schools were provided with information and slides to share with students in advance of the survey.
