# Supplementary material for: Dating and relationship violence victimization and perpetration among 11–16 year olds in Wales: a cross-sectional analysis of the School Health Research Network (SHRN) survey
Source: J Public Health (Oxf). 2019 Aug 29;43(1):111–22. doi: 10.1093/pubmed/fdz084 (PMC8042367; doi:10.1093/pubmed/fdz084)
Supplement: fdz084_Online_supplement_2 [file fdz084_online_supplement_2.docx]

**Online table 1: Prevalence of sociodemographic characteristics of 11-16 year old participants in Wales**

|  | **Sample**  **% (n)** | |
| --- | --- | --- |
| **Sociodemographic characteristics** | **Boys (n=36,419)** | **Girls (n=37,022)** |
| Year group |  |  |
| *Year 7* | 22.4 (8138/36419) | 22.1 (8201/37022) |
| *Year 8* | 21.6 (7871/36419) | 21.8 (8051/37022) |
| *Year 9* | 21.0 (7650/36419) | 21.0 (7772/37022) |
| *Year 10* | 18.7 (6819/36419) | 18.5 (6832/37022) |
| *Year 11* | 16.3 (5941/36419) | 16.7 (6166/37022) |
| Family Affluence Scale (FAS) |  |  |
| *Low* | 34.2 (12462/36419) | 35.1 (13003/37022) |
| *Middle* | 31.5 (11454/36419) | 31.2 (11561/37022) |
| *High* | 34.3 (12503/36419) | 33.7 (12458/37022) |
| Ethnicity |  |  |
| *White British or Irish* | 86.2 (30437/35302) | 87.6 (31429/35890) |
| *White Traveller* | 0.8 (296/35302) | 0.5 (184/35890) |
| *White Other* | 3.2 (1122/35302) | 2.9 (1039/35890) |
| *Mixed Ethnicity or Other* | 4.7 (1670/35302) | 5.1 (1811/35890) |
| *South Asian (Pakistani, Indian, Bangladeshi)* | 2.2 (783/35302) | 1.9 (695/35890) |
| *Chinese* | 0.5 (184/35302) | 0.5 (160/35890) |
| *African or Caribbean or Black* | 1.4 (492/35302) | 1.0 (342/35890) |
| *Arab* | 0.9 (318/35302) | 0.6 (230/35890) |
| Household composition |  |  |
| *Both parents* | 66.1 (19145/28947) | 64.1 (20311/31700) |
| *Single mum* | 16.4 (4737/28947) | 17.9 (5682/31700) |
| *Single dad* | 2.5 (714/28947) | 2.0 (624/31700) |
| *Parent and step-parent* | 13.3 (3861/28947) | 14.9 (4717/31700) |
| *Care* | 1.3 (386/28947) | 1.0 (317/31700) |
| *Other* | 0.4 (104/28947) | 0.2 (49/31700) |
| Ever seeing someone |  |  |
| *Yes* | 55.8 (20333/36419) | 56.1 (20754/37022) |
| *No* | 44.2 (16086/36419) | 43.9 (16268/37022) |
