# Supplementary material for: Dating and relationship violence victimization and perpetration among 11–16 year olds in Wales: a cross-sectional analysis of the School Health Research Network (SHRN) survey
Source: J Public Health (Oxf). 2019 Aug 29;43(1):111–22. doi: 10.1093/pubmed/fdz084 (PMC8042367; doi:10.1093/pubmed/fdz084)
Supplement: fdz084_Online_supplement_3 [file fdz084_online_supplement_3.docx]

**Online table 2: Prevalence of dating experience, emotional victimisation, perpetration and sociodemographic characteristics for the whole sample of 11-16 year old in Wales**

|  | | **Ever seeing someone % (n)** | | **Emotional victimisation % (n)** | | **Emotional perpetration % (n)** | | **Emotional victimisation and perpetration % (n)** | |
| --- | --- | --- | --- | --- | --- | --- | --- | --- | --- |
|  |  | **Boys** | **Girls** | **Boys** | **Girls** | **Boys** | **Girls** | **Boys** | **Girls** |
| **Sex** |  | 55.8 (20333/36419) | 56.1 (20754/37022) | 11.0 (3915/35564) | 15.3 (5565/36440) | 8.9 (3158/35561) | 10.2 (3721/36478) | 7.3 (2594/35517) | 9.1 (3320/36405) |
| **Year** | **7** | 46.7 (3797/8138) | 42.2 (3458/8201) | 6.9 (543/7907) | 6.5 (521/8031) | 4.1 (327/7902) | 3.1 (250/8032) | 3.3 (259/7893) | 2.5 (204/8021) |
|  | **8** | 53.9 (4245/7871) | 52.6 (4231/8051) | 8.4 (642/7667) | 10.0 (791/7916) | 6.4 (488/7666) | 5.4 (428/7918) | 5.0 (385/7654) | 4.5 (357/7903) |
|  | **9** | 58.9 (4507/7650) | 59.6 (4631/7772) | 10.8 (810/7483) | 15.8 (1210/7653) | 8.9 (663/7483) | 10.4 (797/7673) | 7.2 (534/7473) | 9.2 (705/7647) |
|  | **10** | 59.9 (4081/6819) | 62.3 (4257/6832) | 13.6 (907/6663) | 20.3 (1368/6740) | 11.6 (770/6662) | 14.3 (966/6748) | 9.5 (630/6654) | 13.0 (875/6736) |
|  | **11** | 62.3 (3703/5941) | 67.7 (4177/6166) | 17.3 (1013/5844) | 27.5 (1675/6100) | 15.6 (910/5848) | 21.0 (1280/6107) | 13.5 (786/5843) | 19.3 (1179/6098) |
| **FAS** | **Low** | 52.7 (6564/12462) | 55.2 (7177/13003) | 10.6 (1292/12163) | 15.3 (1960/12789) | 8.7 (1058/12158) | 10.4 (1336/12796) | 7.1 (867/12149) | 9.3 (1185/12775) |
|  | **Medium** | 55.8 (6390/11454) | 56.0 (6479/11561) | 11.3 (1262/11177) | 15.0 (1705/11380) | 9.0 (1008/11176) | 10.2 (1164/11398) | 7.5 (836/11158) | 9.1 (1036/11368) |
|  | **High** | 59.0 (7379/12503) | 57.0 (7098/12458) | 11.1 (1361/12224) | 15.5 (1900/12271) | 8.9 (1092/12227) | 9.9 (1221/12284) | 7.3 (891/12210) | 9.0 (1099/12262) |
| **Ethnicity** | **White British or Irish** | 57.5 (17502/30437) | 58.5 (18378/31429) | 11.0 (3265/29768) | 15.9 (4931/30984) | 8.7 (2595/29768) | 10.5 (3258/31020) | 7.2 (2131/29730) | 9.5 (2929/30957) |
|  | **White Traveller** | 69.3 (205/296) | 64.7 (119/184) | 26.2 (73/279) | 30.3 (53/175) | 21.2 (59/279) | 24.6 (43/175) | 19.7 (55/279) | 20.0 (35/175) |
|  | **White Other** | 52.9 (594/1122) | 53.7 (558/1039) | 10.7 (117/1099) | 15.2 (155/1017) | 8.2 (90/1094) | 10.0 (102/1019) | 7.0 (77/1094) | 8.4 (85/1016) |
|  | **Mixed Ethnicity or Other** | 51.0 (851/1670) | 45.8 (830/1811) | 11.3 (185/1636) | 12.8 (227/1779) | 9.3 (152/1639) | 9.0 (161/1781) | 7.1 (116/1635) | 7.8 (138/1777) |
|  | **South Asian (Pakistani, Indian, Bangladeshi)** | 36.1 (283/783) | 26.5 (184/695) | 8.7 (66/761) | 7.6 (52/685) | 9.6 (73/761) | 6.6 (45/684) | 7.8 (59/760) | 6.0 (41/683) |
|  | **Chinese** | 40.8 (75/184) | 37.5 (60/160) | 11.1 (20/180) | 10.8 (17/158) | 8.9 (16/179) | 7.6 (12/157) | 7.8 (14/179) | 6.4 (10/157) |
|  | **African or Caribbean or Black** | 53.9 (265/492) | 38.0 (130/342) | 13.0 (62/478) | 9.5 (32/338) | 13.6 (65/478) | 8.9 (30/338) | 10.9 (52/478) | 6.8 (23/338) |
|  | **Arab** | 40.6 (129/318) | 24.8 (57/230) | 14.1 (43/305) | 4.8 (11/228) | 13.1 (40/305) | 5.7 (13/228) | 11.5 (35/305) | 4.8 (11/228) |
| **Family structure** | **Both parents** | 61.0 (11677/19145) | 57.4 (11667/20311) | 10.8 (2043/18899) | 14.2 (2848/20118) | 8.6 (1619/18892) | 9.2 (1849/20134) | 7.1 (1332/18878) | 8.2 (1638/20102) |
|  | **Single mum** | 66.1 (3132/4737) | 65.9 (3745/5682) | 14.4 (671/4655) | 19.8 (1107/5588) | 11.8 (550/4662) | 13.7 (769/5599) | 9.6 (448/4654) | 12.2 (681/5581) |
|  | **Single dad** | 63.6 (454/714) | 66.7 (416/624) | 15.5 (109/705) | 24.5 (150/613) | 13.2 (93/704) | 18.9 (116/613) | 10.7 (75/703) | 17.7 (108/610) |
|  | **Parent & Step-Parent** | 71.6 (2766/3861) | 73.7 (3478/4717) | 16.5 (625/3795) | 23.3 (1087/4664) | 12.9 (488/3796) | 15.1 (707/4676) | 10.8 (410/3785) | 13.9 (648/4663) |
|  | **Care** | 72.5 (280/386) | 75.7 (240/317) | 23.5 (88/374) | 30.9 (96/311) | 26.0 (97/373) | 26.6 (82/308) | 19.3 (72/373) | 22.7 (70/308) |
|  | **Other** | 65.4 (68/104) | 63.3 (31/49) | 13.7 (14/102) | 16.3 (8/49) | 11.9 (12/101) | 10.2 (5/49) | 8.9 (9/101) | 10.2 (5/49) |
