# Supplementary material for: Dating and relationship violence victimization and perpetration among 11–16 year olds in Wales: a cross-sectional analysis of the School Health Research Network (SHRN) survey
Source: J Public Health (Oxf). 2019 Aug 29;43(1):111–22. doi: 10.1093/pubmed/fdz084 (PMC8042367; doi:10.1093/pubmed/fdz084)
Supplement: fdz084_Online_supplement_4 [file fdz084_online_supplement_4.docx]

**Online table 3: Prevalence of dating experience, physical victimisation, perpetration and sociodemographic characteristics for the whole sample of 11-16 year old in Wales**

|  | | **Physical victimisation % (n)** | | **Physical perpetration % (n)** | | **Physical victimisation and perpetration % (n)** | |
| --- | --- | --- | --- | --- | --- | --- | --- |
|  |  | **Boys** | **Girls** | **Boys** | **Girls** | **Boys** | **Girls** |
| **Sex** |  | 9.5 (3369/35567) | 6.8 (2469/36426) | 3.7 (1311/35579) | 4.7 (1704/36489) | 3.3 (1176/35540) | 3.5 (1267/36404) |
| **Year** | **7** | 7.6 (602/7911) | 4.0 (320/8028) | 2.8 (222/7903) | 2.4 (194/8036) | 2.4 (186/7899) | 1.8 (142/8024) |
|  | **8** | 8.0 (610/7674) | 4.8 (378/7918) | 2.9 (225/7683) | 3.1 (249/7927) | 2.6 (202/7671) | 2.3 (178/7911) |
|  | **9** | 9.6 (719/7481) | 7.0 (533/7650) | 3.7 (280/7483) | 4.9 (373/7676) | 3.3 (245/7474) | 3.6 (276/7647) |
|  | **10** | 10.6 (708/6653) | 8.9 (597/6735) | 4.5 (300/6659) | 6.2 (415/6747) | 4.2 (277/6649) | 4.6 (311/6732) |
|  | **11** | 12.5 (730/5848) | 10.5 (641/6095) | 4.9 (284/5851) | 7.8 (473/6103) | 4.6 (266/5847) | 5.9 (360/6090) |
| **FAS** | **Low** | 9.3 (1133/12164) | 7.1 (912/12782) | 3.6 (440/12164) | 5.0 (636/12808) | 3.2 (391/12155) | 3.7 (467/12776) |
|  | **Medium** | 9.8 (1097/11178) | 6.7 (762/11378) | 3.8 (426/11186) | 4.6 (529/11398) | 3.4 (382/11169) | 3.6 (404/11367) |
|  | **High** | 9.3 (1139/12225) | 6.5 (795/12266) | 3.6 (445/12229) | 4.4 (539/12283) | 3.3 (403/12216) | 3.2 (396/12261) |
| **Ethnicity** | **White British or Irish** | 9.3 (2762/29774) | 6.9 (2138/30972) | 3.3 (994/29789) | 4.7 (1459/31026) | 3.0 (894/29755) | 3.5 (1081/30955) |
|  | **White Traveller** | 25.6 (71/277) | 18.3 (32/175) | 15.8 (44/278) | 15.4 (27/175) | 14.8 (41/277) | 11.4 (20/175) |
|  | **White Other** | 9.3 (102/1097) | 7.4 (75/1016) | 3.5 (38/1096) | 4.6 (47/1016) | 3.0 (33/1095) | 3.5 (35/1014) |
|  | **Mixed Ethnicity or Other** | 9.7 (159/1639) | 6.2 (111/1781) | 4.6 (76/1638) | 4.2 (74/1784) | 4.2 (68/1638) | 3.2 (56/1780) |
|  | **South Asian (Pakistani, Indian, Bangladeshi)** | 8.7 (66/761) | 3.4 (23/685) | 6.4 (49/761) | 2.8 (19/686) | 6.1 (46/760) | 2.2 (15/684) |
|  | **Chinese** | 10.1 (18/178) | 5.1 (8/157) | 5.7 (10/177) | 3.2 (5/157) | 4.0 (7/177) | 3.2 (5/157) |
|  | **African or Caribbean or Black** | 13.8 (66/478) | 5.0 (17/339) | 8.0 (38/478) | 5.6 (19/339) | 7.3 (35/478) | 3.8 (13/339) |
|  | **Arab** | 12.8 (39/305) | 4.8 (11/228) | 8.5 (26/305) | 4.8 (11/228) | 7.5 (23/305) | 4.8 (11/228) |
| **Family structure** | **Both parents** | 8.8 (1663/18896) | 5.8 (1160/20107) | 3.2 (603/18904) | 4.0 (798/20143) | 2.8 (536/18884) | 2.9 (572/20099) |
|  | **Single mum** | 12.3 (571/4654) | 8.8 (493/5593) | 4.4 (205/4656) | 6.3 (354/5606) | 4.0 (185/4651) | 4.8 (269/5590) |
|  | **Single dad** | 13.5 (95/705) | 10.8 (66/609) | 6.1 (43/704) | 8.8 (54/615) | 5.4 (38/704) | 6.1 (37/609) |
|  | **Parent & Step-Parent** | 14.4 (548/3796) | 10.7 (498/4663) | 4.5 (170/3799) | 6.6 (309/4669) | 4.0 (152/3793) | 5.0 (231/4656) |
|  | **Care** | 28.5 (107/375) | 23.5 (72/307) | 23.5 (88/374) | 19.8 (61/308) | 22.2 (83/374) | 17.3 (53/307) |
|  | **Other** | 15.7 (16/102) | 10.2 (5/49) | 11.9 (12/101) | 10.2 (5/49) | 8.9 (9/101) | 8.2 (4/49) |
